# Applying video motion magnification to reveal spontaneous tympanic membrane displacement as an indirect measure of intracranial pressure in patients with brain pathologies

**DOI:** 10.1007/s00701-023-05681-9

**Published:** 2023-06-27

**Authors:** Agnieszka Uryga, Agnieszka Kazimierska, Mateusz Popek, Barbara Dragan, Małgorzata Burzyńska, Marcin Masalski, Magdalena Kasprowicz

**Affiliations:** 1 Department of Biomedical Engineering, Wroclaw University of Science and Technology, Wybrzeże Wyspiańskiego 27, 50-370 Wroclaw, Poland; 2 Department of Anaesthesiology and Intensive Therapy, Wroclaw Medical University, Borowska 213, 50-566 Wroclaw, Poland; 3 Department and Clinic of Otolaryngology, Head and Neck Surgery, Wroclaw Medical University, Borowska 213, 50-566 Wroclaw, Poland

**Keywords:** Tympanic membrane, Video motion magnification, Intracranial pressure, Brain pathology

## Abstract

**Background:**

The observation of tympanic membrane displacement (TMD) opens up the possibility of indirect intracranial pressure (ICP) estimation. In this study, we applied a phase-based video motion magnification (VMM) algorithm to reveal spontaneous pulse TMD waveforms (spTMD) and compare them with invasively measured ICP in patients with intracranial pathologies.

**Methods:**

Nine adults (six traumatic brain injury and three aneurysmal subarachnoid haemorrhage; median age 44 (29–53) years admitted to the intensive care unit of Wroclaw Medical University between October 2021 and October 2022 with implanted ICP sensors were included in this retrospective study. Video recordings of the tympanic membrane were performed using a portable otoscope with a video camera and analysed by a custom-written VMM algorithm. ICP was monitored using intraparenchymal sensors and arterial blood pressure (ABP) was measured in the radial arterial lines. ICP, ABP, and spTMD videos were captured simultaneously. The pulse amplitudes of ICP (Amp_ICP), ABP (Amp_ABP) and spTMD (Amp_spTMD) were estimated using fast Fourier transform within the heart rate (HR)–related frequency range.

**Results:**

Amp_spTMD was significantly correlated with mean ICP (*r*_*S*_ = 0.73; *p* = 0.025) and with Amp_ICP (*r*_*S*_ = 0.88; *p* = 0.002). Age was not a significant moderator of this association. There were no significant relationships between Amp_spTMD and either mean ABP, HR, or Amp_ABP.

**Conclusions:**

The study suggests that Amp_spTMD increases with the increase in mean ICP and Amp_ICP. Estimation of Amp_spTMD using the VMM algorithm has the potential to allow for non-invasive detection of the risk of elevated ICP; however, further investigation in a larger group of patients is required.

## Introduction

Currently, invasive intracranial pressure (ICP) monitoring is restricted to patients with severe brain injury for whom the risk of serious complications associated with ICP sensor implantation is outweighed by the need for timely detection of raised ICP [9]. Increased ICP may cause impaired cerebral perfusion leading to brain ischemia or herniation of brain tissue and may result in death [6]. Although ICP monitoring in acute intracranial pathologies is deemed essential by evidence-based guidelines [4], in low-income countries the management of intracranial hypertension is mostly based on radiological assessment as ICP sensors are not widely available [10, 31]. Therefore, alternative means of estimating ICP in a reliable but low-cost and non-invasive manner are needed.

One of the possible approaches to gathering information about the ICP waveform is to study the cochlear aqueduct which transmits the pressure pulsation from the intracranial compartment to the perilymphatic space of the inner ear. The infrasonic ICP waveforms cause the oscillations of the oval window and ossicles, consequently leading to spontaneous tympanic membrane displacement (spTMD) [9]. The conventional technique for spTMD assessment requires sealing of the ear in an air-tight fashion for the displacements to be detected, which is challenging due to the anatomical variability of the ear canal [19]. Another way to assess ICP is to measure TMD triggered by the stapedial reflex following acoustic stimulation [13, 26]. This approach, however, does not allow for continuous spTMD monitoring.

A promising method that may overcome the limitations of the conventional techniques is to collect video recordings of the tympanic membrane and apply image processing methods to reveal the membrane motions that are otherwise undetectable by the human eye. Video motion magnification (VMM) algorithms that amplify spatiotemporal changes between consecutive frames of the video have been successfully used to reveal the subtle motion of the tympanic membrane in earlier studies [22]. However, little evidence is available about the reliability of using VMM-supported TMD assessment in periods of elevated ICP which may occur in patients with intracranial pathologies.

In this paper, a phase-based VMM technique [24] was employed to reveal spTMD without sealing the ear canal. This study aimed to explore whether the spTMD waveform related to the cardiac cycle can be used to non-invasively estimate the ICP pulse waveform in patients with intracranial pathologies.

## Materials and methods

### Settings and ethics

The study conforms to the Declaration of Helsinki, and the research protocol was approved by the Bioethics Committee at the Medical University in Wroclaw, Poland (approval no. KB-620/2020, KB-801/2020, KB-132/2023), which waived the requirement for informed consent based on the study design.

### Study population

This retrospective, single-centre, observational study was conducted at the Intensive Care Unit of the University Clinical Hospital in Wroclaw, Poland, from October 2021 to October 2022. The group included nine adult patients in whom a parenchymal ICP sensor was implemented: six with a diagnosis of traumatic brain injury (TBI) and three with aneurysmal subarachnoid haemorrhage (aSAH). The group consisted of two women and seven men with a median age of 44 (29–53) years. Based on the Glasgow coma scale (GCS) assessment, the clinical condition of most patients was severe: severe (scores 3–8) — seven subjects; moderate (scores 9–12) — one subject; mild (scores 13–15) — one subject. Median body mass index (BMI) was 26.1 (22.9–27.7). Detailed study group characteristics are presented in Table 1. Included patients had no signs of ear bleeding nor perforation of the tympanic membrane, and in TBI patients, basal skull fractures or skull fractures involving the temporal bone were excluded.

**Table 1:** Patient characteristics and the mean value of physiological signals, including the spectral amplitude of spontaneous tympanic membrane displacement (spTMD). As the values for each patient were calculated from a time window lasting tens of seconds, the variability is not presented

No	cat	Age [years]	Sex	GCS	Amp_spTMD [μm]	HR [bpm]	ICP [mm Hg]	Amp_ICP [mm Hg]	ABP [mm Hg]	Amp_ABP [mm Hg]
1	aSAH	53	M	13	11.3	63	24.7	5.2	81.5	18.1
2	TBI	26	M	8	6.7	74	14.5	2.8	95.9	14.0
3	TBI	24	K	5	2.8	105	11.3	1.0	80.0	13.9
4	aSAH	44	K	5	3.1	113	7.4	0.8	82.3	16.9
5	TBI	62	M	8	5.7	67	18.9	4.5	78.3	19.7
6	TBI	29	M	8	2.7	86	5.0	1.0	70.9	18.6
7	aSAH	61	M	4	3.4	61	10.0	2.0	83.3	21.9
8	TBI	45	M	10	2.8	46	9.9	1.4	87.0	24.0
9	TBI	33	M	3	4.0	57	9.0	2.5	79.2	17.0

*cat.* Patient’s diagnosis; *aSAH* aneurysmal subarachnoid haemorrhage; *TBI* traumatic brain injury; *GCS* Glasgow coma scale; *ICP* intracranial pressure; *Amp_ICP* spectral amplitude of ICP; *ABP* arterial blood pressure; *Amp_ABP* spectral amplitude of ABP; *Amp_spTMD* spectral amplitude of spontaneous tympanic membrane displacement, *HR* heart rate

### Data acquisition

**Fig. 1:**
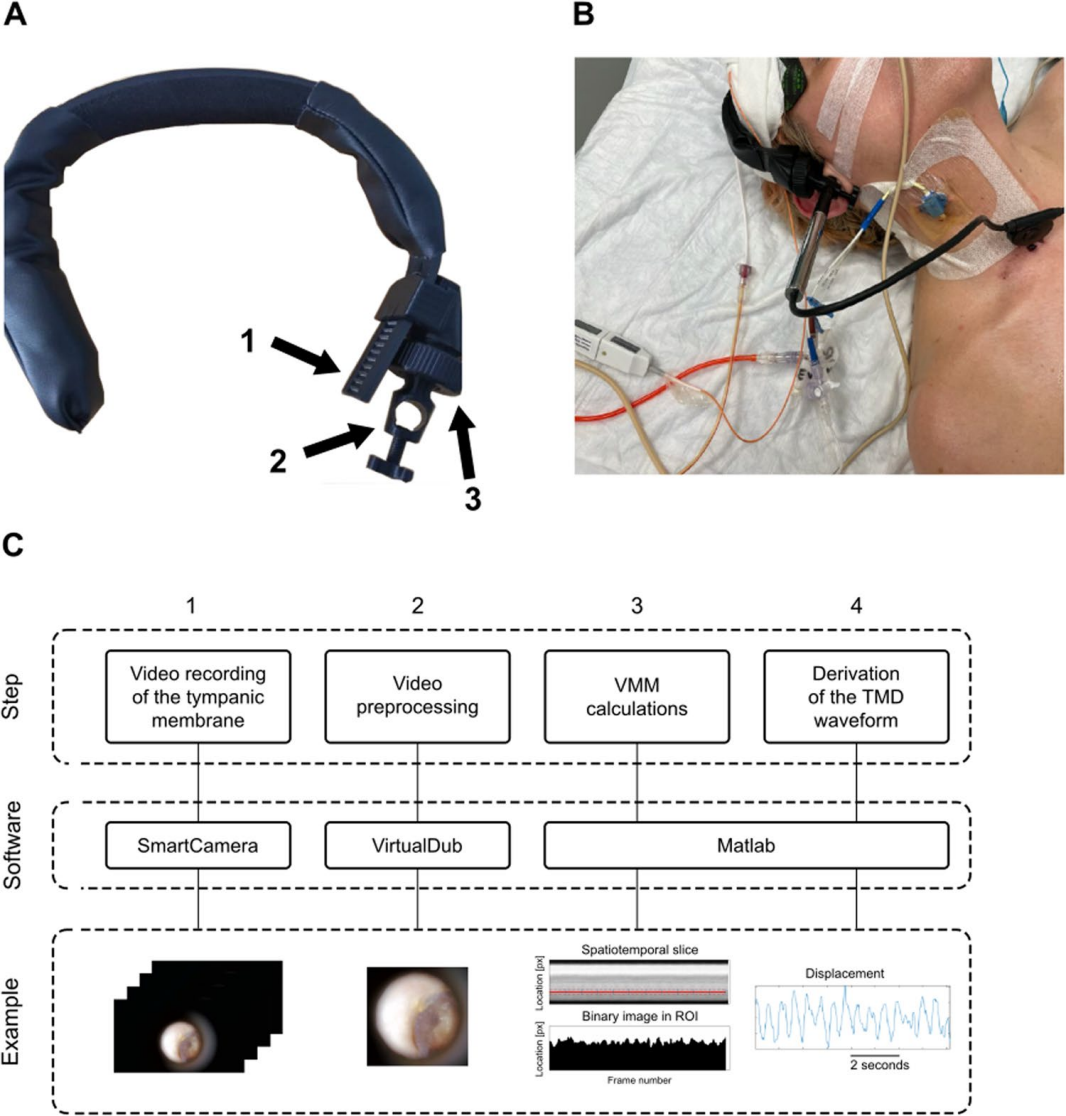
**A** Otoscope fixation system. The portable videoscope was stabilized during measurements to avoid minor movements or loss of focus using a custom-made fixation system created with the 3-D printer from polylactic acid (PLA): 1–adjustment of band circumference, 2–hole for the otoscope with a stabilizing screw at the bottom, 3–ball joint for adjusting the otoscope angle. **B** Measurement of spontaneous tympanic membrane displacement (spTMD) in a patient with traumatic brain injury. **C**. The video processing pipeline. Step 1 A 20-s-long video recording of the tympanic membrane was performed using a portable videoscope and SmartCamera software during simultaneous measurement of arterial blood pressure (ABP) and intracranial pressure (ICP). The start and end of the video were annotated in ICM + software to enable synchronization of spTMD, ICP, and ABP recordings. Step 2. The video was pre-processed (cropping and trimming the length of the video) using VirtualDub software. Steps 3 and 4. Lastly, the video was processed using the video motion magnification (VMM) algorithm in Matlab to reveal spTMD over time

**Fig. 2:**
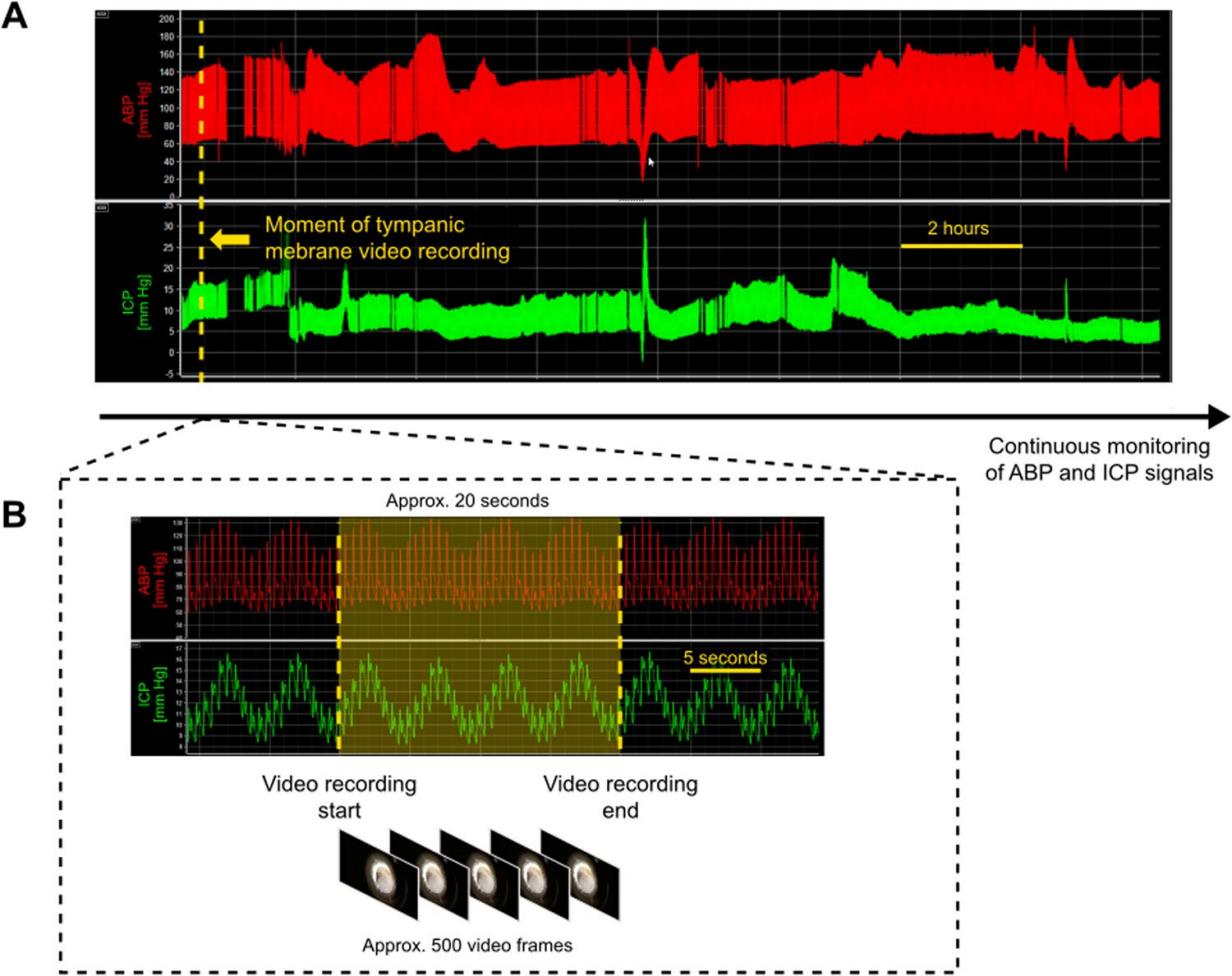
Synchronization between intracranial pressure (ICP) and arterial blood pressure (ABP) signal recordings and ‘snapshot’ video recordings of the tympanic membrane. **A** ICP and ABP were monitored continuously throughout the patient’s entire stay in the intensive care unit. At a time point selected by the intensivist (yellow dashed line), a video recording of the tympanic membrane was collected. **B** The video recording of the tympanic membrane lasted approx. 20 s (approx. 500 frames at 25 frames per second). The start and end of the recording were marked by the clinician (yellow dashed lines). The video recording was processed separately to extract spontaneous tympanic membrane displacement signal (see Fig. 3). The marked fragment of the ICP and ABP signal recordings (yellow shaded area) was used to calculate signal metrics corresponding to the time when the video was taken in order to assess if instantaneous ICP-derived parameters are reflected in tympanic membrane displacement

Two signals were continuously measured in each patient: arterial blood pressure (ABP) using a radial arterial line (Baxter Healthcare Corp., Irvine, CA), and ICP using an intraparenchymal sensor (Codman & Shurtleff, MA, USA). ABP and ICP were recorded with a sampling frequency of 200 Hz using the Intensive Care Monitor (ICM +) system (Cambridge Enterprise Ltd, Cambridge, UK). The video recording of the tympanic membrane was captured as a video ‘snapshot’ lasting approximately 20 s using a portable videoscope (Hearscope, HearX group, Pretoria, South Africa) with 2-megapixel CMOS sensor with 1920 × 1080 resolution and a framerate of 25 fps. The video was recorded on a portable laptop using SmartCamera software. During the measurements, a standard ear speculum attached to a custom-made fixation system (see Fig. 1A–B) was used to stabilize the videoscope in order to avoid minor movements or auto-focus adjustments. The start and end of the video were annotated in ICM + software to enable synchronization of spTMD, ICP, and ABP recordings, see Fig. 2. Then, values of ICP and ABP were taken from signals at exactly the same moment of time when a short-time video of the tympanic membrane was performed.

### Data processing

VMM analysis was performed using a custom-written algorithm [23] based on open-source code [30] using MATLAB R2020a (MathWorks, Natick, MA, USA). The algorithm was developed specifically for motion-magnified videos and is mostly resistant to variations in brightness and contrast. The VMM method consists of three main steps. Firstly, a video frame is divided into parts with different spatial scales and orientations using a complex steerable pyramid with a given filter complexity to separate local amplitudes from phases. Secondly, the signal is filtered to extract band-passed single phases, and phase changes are amplified by the empirically selected factor of *α* = 20. Lastly, each frame is reassembled from the pyramids, producing a video with amplified motion. Motions are magnified with regard to a chosen reference frame of the video. A detailed description of the VMM algorithm workflow can be found in our previous paper [24].

In this study, recorded videos were about 20 s long as the processing is computationally intensive (taking about 10 min to process 20 s of video consisting of about 600 × 660 pixels of the frame section on a modern Intel i7 computer). During the measurements, a constant distance (25 mm) was maintained between the videoscope and the tympanic membrane by setting the maximum image sharpness for the given distance (constant focus plane). Recalculation of the displacement expressed in pixels to values expressed in micrometres was performed using a calibration curve which was constructed based on micrometre displacements of a metal plate affixed to a speaker membrane driven by a function generator. These displacements were measured using an air-coupled ultrasonic transducer (UltraLab, Wroclaw, Poland) with video recordings processed with the VMM method captured in parallel. Details of the calibration procedure are available in our previous paper [23]. In each recording, the spTMD was estimated in a manually selected region (A–I; anterior–inferior) of the tympanic membrane. The algorithm of tympanic membrane video processing is presented in Fig. 1C.

The spectral amplitude of ICP (Amp_ICP), ABP (Amp_ABP), and spTMD (Amp_spTMD) were estimated using fast Fourier transform in the heart rate–related frequency range (0.66–3.0 Hz). Heart rate (HR) was calculated using the spectral position of the peak associated with the first harmonic of ABP.

### Statistics

Normality of the data was assessed using the Shapiro–Wilk test. Because of the rejection of the normality hypothesis for most of the analysed parameters and limited number of observations, non-parametric tests were applied. Correlation between numerical data was assessed using Spearman’s rank correlation coefficient (r_S_). Multiple regression was run to predict mean ICP from Amp_spTMD and age; the impact of individual factors is reported using the regression coefficient (β). The level of significance was set at 0.05 in all analyses. Statistical analysis was performed using R Statistical Software (v.4.0.2; R Foundation for Statistical Computing, Vienna, Austria). Data are presented as median (first–third quartile) unless indicated otherwise.

## Results

### Spontaneous tympanic membrane displacement

**Fig. 3:**
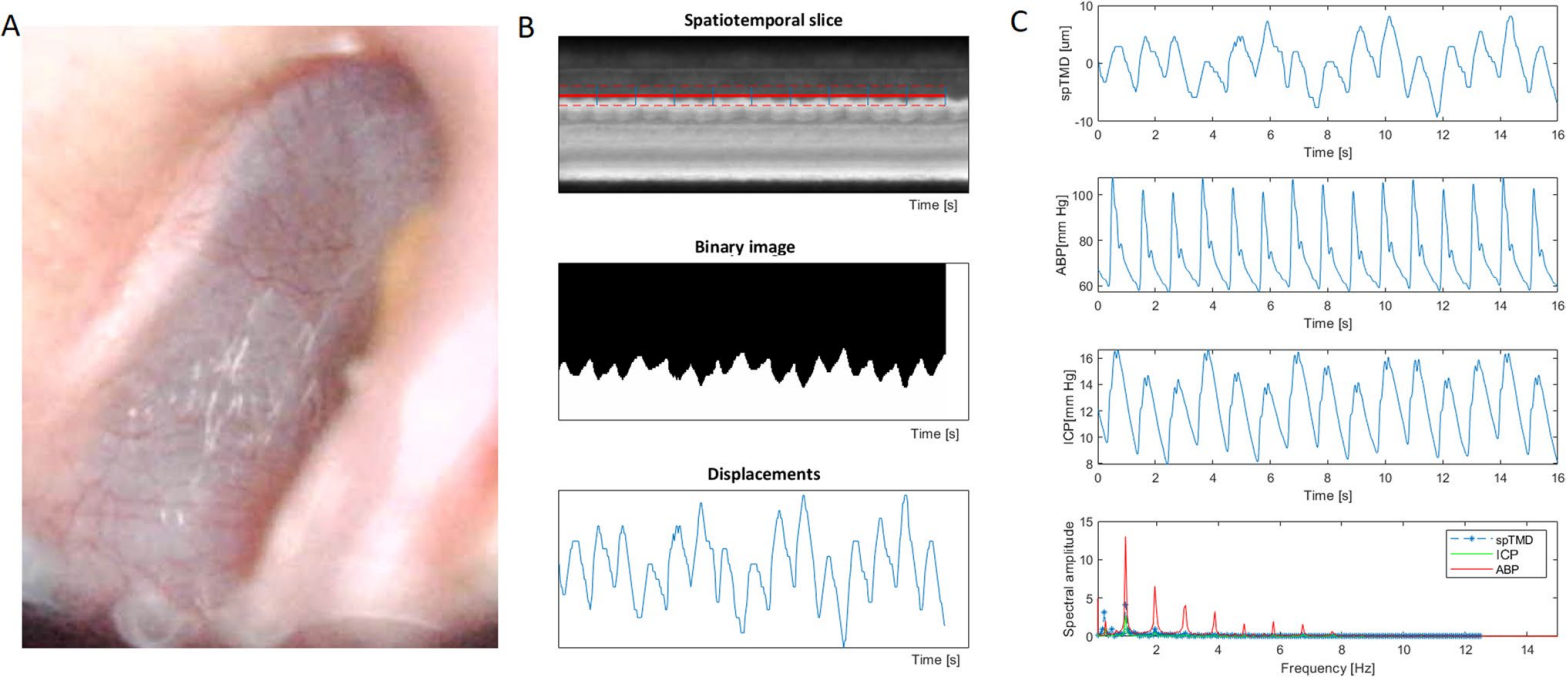
**A** An illustrative example of a video frame from the tympanic membrane recording in a traumatic brain injury patient. **B** Intermediate steps to obtain the displacement waveform from a video frame. **C** The time course of spontaneous tympanic membrane displacement (spTMD), arterial blood pressure (ABP), and intracranial pressure (ICP), and the amplitude spectrum of all three signals

**Fig. 4:**
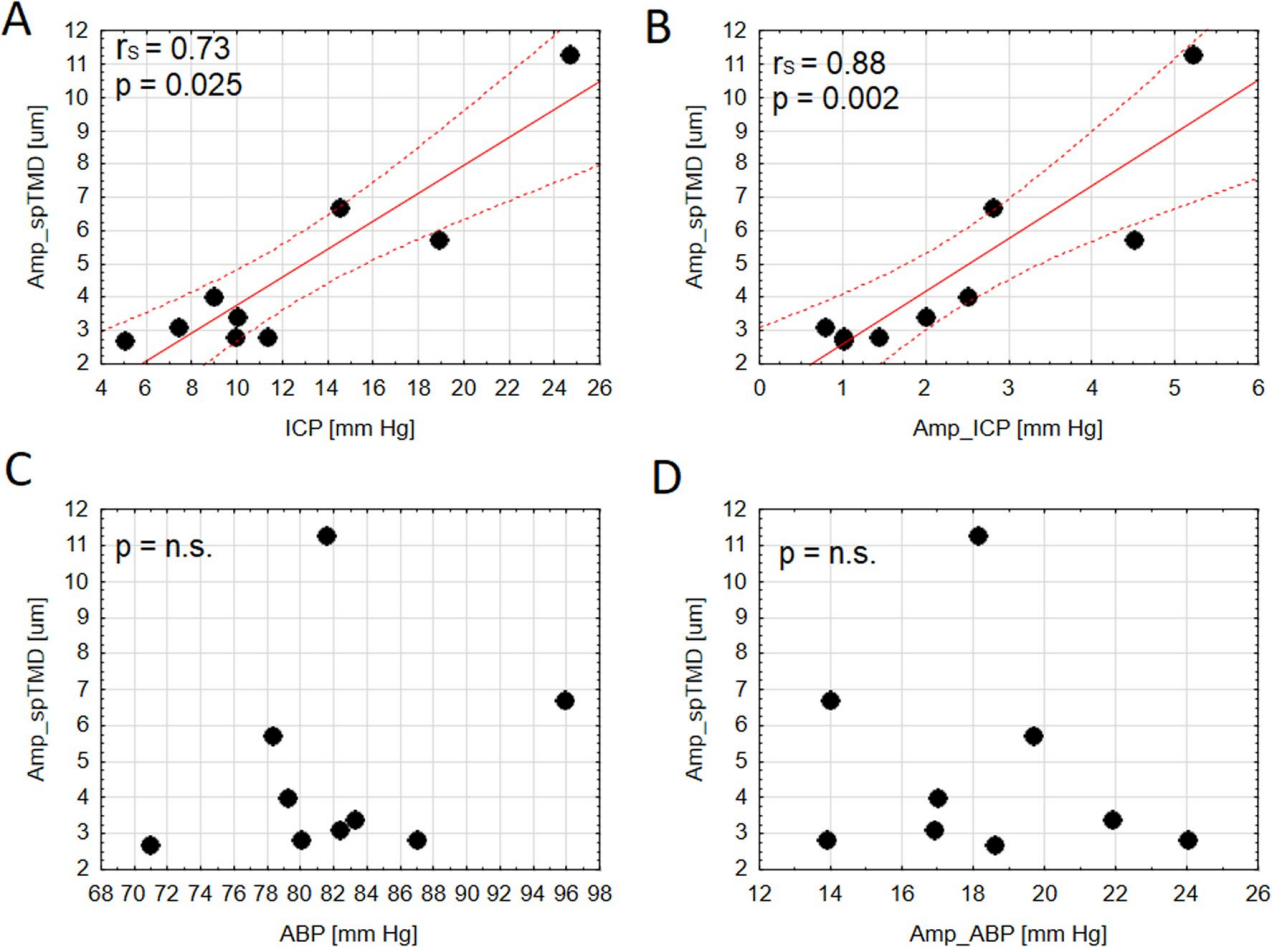
The correlation plots for the spectral amplitude of spontaneous tympanic membrane displacement (spTMD) and **A** mean intracranial pressure (ICP), **B** spectral amplitude of intracranial pressure (Amp_ICP), **C** arterial blood pressure (ABP), and **D** spectral amplitude of ABP (Amp_ABP). The regression line (solid line) and 95% of predictor limits (dashed lines) are shown in Figures A–B; *r*_*s*_ — nonparametric Spearman correlation coefficient

In the total group, the average Amp_spTMD was 3.4 (2.8–5.7) μm. An example of spTMD waveform in high ICP conditions is presented in Fig. 3. The frequency of the first harmonic of spTMD was consistent with the frequency of the fundamental component of ICP and ABP, see Fig. 3C. In the group analysis, Amp_spTMD was strongly correlated with mean ICP (*r*_*S*_ = 0.73; *p* = 0.025) and Amp_ICP (*r*_*S*_ = 0.88; *p* = 0.002), see Fig. 4A–B. Conversely, no significant relationship between Amp_spTMD and either mean ABP (*p* = 0.488), HR (*p* = 0.460), or Amp_ABP was found (*p* = 0.865), see Fig. 4C–D.

### Spontaneous tympanic membrane displacement vs. age

Multiple regression was run to predict mean ICP from Amp_spTMD and age. These variables statistically significantly predicted ICP, F (2, 6) = 6.69, *p* = 0.030, *R*^2^ = 0.58. Amp_spTMD was positively related to mean ICP (*β* = 0.86, *p* = 0.002); however, age did not significantly moderate the effect of mean ICP (*β* = 0.19, *p* = 0.262).

## Discussion

The results of the present study confirmed the feasibility of using advanced techniques of video processing to reveal spontaneous pulsatile changes in TMD. Furthermore, we showed that the frequency of the first harmonic of spTMD is congruent with the frequency of the first harmonic in ICP and ABP signals. This indicates that the pulsatile character of all of those signals is related to the cardiac cycle. We also found that elevated mean ICP and Amp_ICP are related to higher Amp_spTMD estimated from motion-magnified videos. Notably, Amp_spTMD was not correlated with either mean ABP or Amp_ABP. Therefore, we concluded that the oscillations revealed in the tympanic membrane are a result of pulse changes in ICP. This observation is consistent with the findings of El-Bouri et al. [7] who demonstrated that the power of the spTMD signal is contained mostly in the physiological frequency ranges related to respiration (and its harmonics) and HR (and its harmonics). They also showed that information shared between the ICP and spTMD signals is not present in ABP [7]. In our study, we did not observe a significant relationship between sp_TMD and either ABP or HR; however, in the analysed group of patients, the variability of ABP was relatively low (min–max: 70–95 mm Hg) as the optimal cerebral perfusion pressure (CPP) was the treatment target. Therefore, a further study including a broader range of ABP (or CPP) is needed. In our study, the Amp_spTMD was not related to age, which is in line with the study about ageing in the human cochlear aqueduct [12].

To date, invasive ICP monitoring remains the most accurate method of detecting intracranial hypertension. However, the intrusive character of this approach, along with the associated risk of complications and high treatment costs, limit its applicability. Therefore, there is a crucial need for non-invasive ICP estimation to enable early detection of intracranial hypertension. Various studies have explored the use of ABP waveforms as source signals for non-invasive ICP [8, 16]. Additionally, the ABP signal has been combined with other non-invasively obtained signals, such as transcranial Doppler–derived cerebral blood flow velocity, to estimate ICP [14, 17, 27]. Other proposed non-invasive methods of ICP monitoring include modelling of fluid dynamics within the intracranial space, ophthalmic, otic, and electrophysiological measurements, magnetic resonance imaging, near-infrared spectroscopy, and optical coherence tomography of the retina [34]. However, none of these methods have achieved sufficient levels of accuracy to be widely accepted in the clinical setting. Furthermore, the primary focus of most methods proposed to date has been to estimate mean ICP level rather than the full ICP waveform. In recent years, a different method utilising mechanical extensometers placed in contact with the surface of the skull that was proposed by Mascarenhas et al. [21] and implemented in the Brain4care® device [2, 3, 11] started receiving more attention. This technology enables non-invasive estimation of ICP morphology and provides information about brain compliance based on characteristic peaks P1 and P2 of the ICP pulse waveform [2, 3, 11].

Analysis of TMD is another approach for tracking ICP and intracranial compliance in neurosurgical patients. The original approach to TMD measurement, pioneered by Marchbanks et al. [25] and based on mechanical sensors placed within a sealed ear canal, identified certain characteristics of the intra-aural pressure pulse that could be interpreted as surrogates for characteristic waveforms in ICP [13, 26]. Lang et al. [19] demonstrated the utility of this approach in the assessment of cerebrospinal compliance. They compared the high-frequency centroid calculated from an intraparenchymal ICP sensor with that obtained simultaneously from an ipsilaterally placed non-invasive TMD sensor during three heart cycles. Their results showed that intracranial compliance assessment based on the high-frequency centroid of ICP estimated using TMD waveform is equivalent to that obtained by invasive intraparenchymal recording.

However, the studies about the impact of ICP on TMD are inconclusive. It was found that the alterations in ICP can affect the hydrostatic pressure of the cochlea and influence the peripheral auditory system [25], but transmission from the intracranial cerebrospinal fluid spaces to the middle ear depends on the patency of the cochlear aqueduct [5]. Therefore, it has been hypothesised that elevated ICP may cause an increase in the amplitude of the TMD waveform [19]. It has been shown that the TMD test can be used as a reproducible investigative tool in the assessment of ICP in many clinical scenarios, such as adult and paediatric hydrocephalus shunting, intracranial tumours, and other neurological conditions associated with abnormal ICP [25, 26, 29], which may significantly reduce the need for invasive ICP monitoring. On the other hand, the paper of Evensen et al. [9] has demonstrated that the tympanic membrane pressure waveforms measured in the outer ear could not sufficiently estimate pulse ICP measures, and the cochlear aqueduct may work as a physical low-pass filter. In another study by [12], it was stated that in normal ears, the cochlear aqueduct filters out cardiac and respiration-induced pulses from cerebrospinal fluid and prevents them from affecting the cochlear function.

Our approach is based on the relatively new method in biomedicine known as VMM which allows us to reveal micrometre-scale movements [32]. The VMM techniques were applied successfully as a relatively low-cost modality for measuring small motions in different fields of biomedicine: in ophthalmology to reveal the corneal pulse [23], in medical imaging to visualize local variations in the stiffness of cardiovascular tissue [22], in posturography to improves the clinician’s ability to identify atremulous hands as Parkinsonian [32], and in analysis of microscopic videos of biological cells [28]. VMM was also applied to improve free flap monitoring during surgery [20] and as a video-optimizing approach in endoscopic and laparoscopic surgery [1]. In laryngology, it was shown by Won et al. that the phase-based motion magnification of unsealed pneumaticotoscopy reveals comparable eardrum motions as in standard pneumatic otoscopy with a sealed ear canal [33]. Moreover, Janatka et al. presented the possibility of diagnosing otitis media with effusion using a smartphone videoscope and phase-based VMM [15]. However, the usefulness of VMM for revealing pulse TMD changes in relation to different ICP levels has not been analysed before. The proposed imaging-based approach for ICP screening is cheaper than the traditional invasive technique and may be used not only in hospital wards but also in specialist clinical practices. It was demonstrated in previous research that the shape of the TMD pulse waveform measured by conventional techniques changes when ICP increases or/and intracranial compliance deteriorates [19]. Therefore, the next step should include morphological analysis of the spTMD waveforms revealed with the VMM approach.

While most studies on the relationship between TMD and ICP focus on acoustic stimulation of the stapedial reflex causing movement of the tympanic membrane using pneumatic techniques and mechanical sensors [33], we proposed a methodology that relies on simple measurement done via a portable otoscope with a high-resolution camera. A single, short video lasting tens of seconds is processed by the VMM algorithm that allows for the determination of cardiac-related spTMD waveform without any external stimulation of the tympanic membrane and without the need for ear sealing. Ultimately, the findings of this study suggest that that VMM method could allow for spTMD tracking in patients with intracranial pathologies and provide the means to identify patients with elevated ICP. The proposed approach seems encouraging as it could be easily applied in routine clinical use with relatively cheap and widely available tools (i.e., a videoscope and a computer), therefore, allowing for quick screening of patients at risk of intracranial hypertension. Nonetheless, at present, our method is not suitable for accurate tracking of the morphological changes in spTMD, and it cannot be confirmed that characteristic peaks P1, P2, and P3 (i.e., the percussion, tidal, and dicrotic waves) in the spTMD waveform are similar to the ICP signal. Moreover, as crucial information is provided not only by mean ICP value but also by its dynamic changes in time, further investigations are needed to develop a method for long-time spTMD recording. If similar time trends will be observed in ICP and spTMD for a longer period of time, it would more accurately demonstrate the potential of the proposed method of utilising spTMD as ICP surrogate. Further studies are required to assess the efficacy of morphological analysis of TMD pulse waveform as a tool to estimate cerebrospinal compliance.

Finally, several limitations deserve attention. This study was performed on a small number of observations due to the limited availability of ICP measurements in the study centre associated with COVID-19 pandemic restrictions. This approach is also limited by laryngological malformations that occur in patients with intracranial pathologies: obstruction of the ear canal, craniofacial bone fractures, and tympanic membrane perforation. Taking into account the limited number of included patients and the fact that only a few of them presented high ICP, the correlation results may be biased due to the characteristics of the low ICP group; therefore, more data are needed to confirm the results of this preliminary study. Furthermore, measurements in the neurocritical care environment are difficult due to mechanical ventilation which may influence the estimated spTMD. The spTMD was estimated in an arbitrarily selected region (A–I; anterior–inferior) of the tympanic membrane. It has been shown in a previous study that the superior regions of the eardrum undergo greater displacements than the region near the light reflex [25]. Moreover, the dynamic motions of the tympanic membrane are complex and contain lateral and axial movements [25]. The influence of the selected tympanic membrane area on obtained spTMD waveform was not investigated. The videoscope measurements were performed in one ear, and we did not assess possible differences between the sides of the head. Moreover, high intra-patient variability needs to be considered in further investigations as the anatomical conditions such as the individual configuration of the cochlear aqueduct may influence the transmission of the waveforms to the tympanic membrane. Lastly, in this analysis, we did not study the full TMD waveform recording but used it to derive a single value which served as a surrogate of instantaneous measurement. VMM is computationally intensive; therefore, at the moment only short videos are suitable for processing without specialized workstations. However, this limitation could be overcome by using VMM based on machine learning methods that are currently under development [18]. Moreover, it seems rational to repeat measurements during an infusion test, during which significant changes in ICP occur over a short period of time.

## Conclusions

Our results demonstrate the potential of using the VMM technique to screen patients with intracranial pathologies for the risk of intracranial hypertension. This approach could be used as a low-cost diagnostic method of screening for intracranial hypertension even in low-income environments provided a modern computer is provided. Further work on a larger group of patients is needed to confirm these observations.
